# Mechanisms of alcohol influence on fear conditioning: A computational model

**DOI:** 10.1111/acer.70071

**Published:** 2025-05-19

**Authors:** Adam Lonnberg, Marian L. Logrip, Alexey Kuznetsov

**Affiliations:** ^1^ Cleveland Clinic Neurology Residency Cleveland Ohio USA; ^2^ Department of Psychology Indiana University Indianapolis Indianapolis Indiana USA; ^3^ Department of Mathematical Sciences Indiana University Indianapolis Indianapolis Indiana USA

**Keywords:** amygdala circuit, computational model, electrophysiological activity, fear acquisition, fear extinction

## Abstract

**Background:**

A connection between stress‐related illnesses and alcohol use disorders is extensively documented. Fear conditioning is a standard procedure used to study stress learning and links it to the activation of amygdala circuitry. However, the connection between the changes in amygdala circuitry and function induced by alcohol and fear conditioning is not well established.

**Methods:**

We introduce a computational model to test the mechanistic relationship between amygdala functional and circuit adaptations during fear conditioning and the impact of acute vs. repeated alcohol exposure. Using firing rate formalism, the model generates electrophysiological and behavioral responses in fear conditioning protocols via plasticity of amygdala inputs. The influence of alcohol is modeled by accounting for known modulation of connections within amygdala circuits, which consequently affect plasticity. Thus, the model connects the electrophysiological and behavioral experiments. We hypothesize that alterations within amygdala circuitry produced by alcohol cause abnormal plasticity of amygdala inputs such that fear extinction is slower to achieve and less robust.

**Results:**

In accordance with prior experimental results, both acute and prior repeated alcohol decrease the speed and robustness of fear extinction in our simulations. The model predicts that, first, the delay in fear extinction caused by alcohol is mostly induced by greater activation of the basolateral amygdala (BLA) after fear acquisition due to alcohol‐induced modulation of synaptic weights. Second, both acute and prior repeated alcohol shift the amygdala network away from the robust extinction regime by inhibiting activity in the central amygdala (CeA). Third, our model predicts that fear memories formed during acute or after chronic alcohol are more connected to the context.

**Conclusions:**

The model suggests how circuit changes induced by alcohol may affect fear behaviors and provides a framework for investigating the involvement of multiple neuromodulators in this neuroadaptive process.

## Graphical abstract

Computational models can test mechanistic relationships between fear conditioning, neuroadaptations, and alcohol. Simulated fear extinction is slower and less robust under alcohol, as greater basolateral amygdala activation is needed. Alcohol shifts the amygdala network away from robust extinction by inhibiting central amygdala activity. Context may play a larger role in fear memories formed during acute or after chronic alcohol. The model demonstrates how alcohol‐induced circuit changes affect fear learning, providing a framework for investigating neuromodulators' effects in this neuroadaptive process.

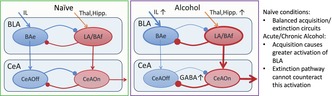

## INTRODUCTION

Alcohol use disorder (AUD) is a chronic condition characterized by loss of control over drinking and a high propensity to relapse despite periods of abstinence (NIAAA, 2021). Stress is a predisposing factor for AUD and other mental illnesses, and past trauma increases the propensity to develop AUD, as well as being a primary relapse trigger (Moos & Moos, 2007). This suggests similar neurocircuits underlie the maladaptive effects of stress and AUD. Activation of stress circuitry not only contributes to AUD pathology (Koob, 2013) but likely regulates stress potentiation of AUD development. Among stress‐related mental illnesses, posttraumatic stress disorder (PTSD) is characterized by rapid reacquisition of the fear response following a generally mild stressor, and AUD often potentiates these symptoms (Saladin et al., 1995). Elucidating at the circuit level how stress and alcohol impact neurons and the implications for behavior is important for identifying novel treatments for comorbid stress‐ and alcohol‐related diseases.

Fear conditioning has long been studied to elucidate how stress learning alters neural circuits in rodents (Ressler & Maren, 2019). Studies focus on the initial learning of the fear behavior (acquisition), pairing a conditioned stimulus (CS; cue or context) with an unconditioned stimulus (US; the stressor), and on the processes by which fear‐related behaviors fade over repeated exposure to the CS in the absence of the US (extinction). Extinction may be permanent or transient (Rescorla, 2004; Rescorla & Heth, 1975), depending on the strength of physiological changes in the stress circuitry during the formation of the extinction memory (Courtin et al., 2014). Fear can reappear due to multiple factors, including the passage of time, a change in context, or the reappearance of the US (Williams & Lattal, 2019). These studies serve as an experimental tool for behavioral research on the interaction between stress and alcohol effects in rodents and other model organisms.

Stress‐alcohol interactions are complex and incompletely understood. Single or repeated fear conditioning sessions prior to alcohol exposure increase future alcohol intake (Logrip & Zorrilla, 2012; Meyer et al., 2013). Acute alcohol can impair (Dickerson & Ferraro, 1976; Williams & Lattal, 2019), produce no effect (Bisby et al., 2015; Kitaichi et al., 1995), or even amplify fear acquisition at low alcohol doses (Gulick & Gould, 2007). While these inconsistencies likely are driven by differences in the temporal presentation of stress and alcohol or the frequency of stress exposure (Becker et al., 2011), they may be subtle and challenging to consistently model. Conversely, fear extinction learning is consistently slower and weaker under acute alcohol exposure (Bisby et al., 2015; Lattal, 2007).

Chronic alcohol has variable effects on fear acquisition: facilitation (Bertotto et al., 2006) or no effect (Holmes et al., 2012) in rodents and reduction in humans (Stephens et al., 2005). Chronic alcohol also facilitated expression of preexisting fear memories (Quiñones‐Laracuente et al., 2015). Binge drinkers slowed fear acquisition but generalized reactions to fear and nonfear CS (Stephens et al., 2005). However, fear extinction is consistently weaker after chronic alcohol exposure (Bertotto et al., 2006; Holmes et al., 2012; Smiley et al., 2020). Here, we aim to establish mechanistic relationships between altered behavioral functions and structural adaptations of brain circuits during fear learning under acute and chronic alcohol.

### Circuits for alcohol‐stress interactions

We connect alcohol‐induced alterations at the circuit and behavioral levels in a computational model. The amygdala plays a central role in fear learning (Plas et al., 2024). Potentiating connections between amygdalar nuclei and their inputs via synaptic plasticity drives fear acquisition, particularly at thalamic connections to the lateral amygdala (LA) and at hippocampal projections to the basal nucleus (BA) (Herry et al., 2008; Pare & Duvarci, 2012). Rather than reversing acquisition‐related changes, fear extinction is new learning that strengthens different amygdala inputs to suppress the activity of fear‐encoding neurons (Moustafa et al., 2013; Williams & Lattal, 2019). Every fear conditioning test reactivates the conditioned fear memory despite the absence of the US as part of the active maintenance of the fear memory (Nader, 2015). This should not be confused with the new learning that underlies extinction of fear‐conditioned behavior. Multiple CS presentations without US exposure are required to generate the new learning underlying fear response suppression, that is, extinction learning (Ferrara et al., 2023), by potentiating cortical inputs to a subset of BA neurons. To construct our model, we integrate data on core components of the amygdala.

#### Basolateral amygdala

**FIGURE 1:**
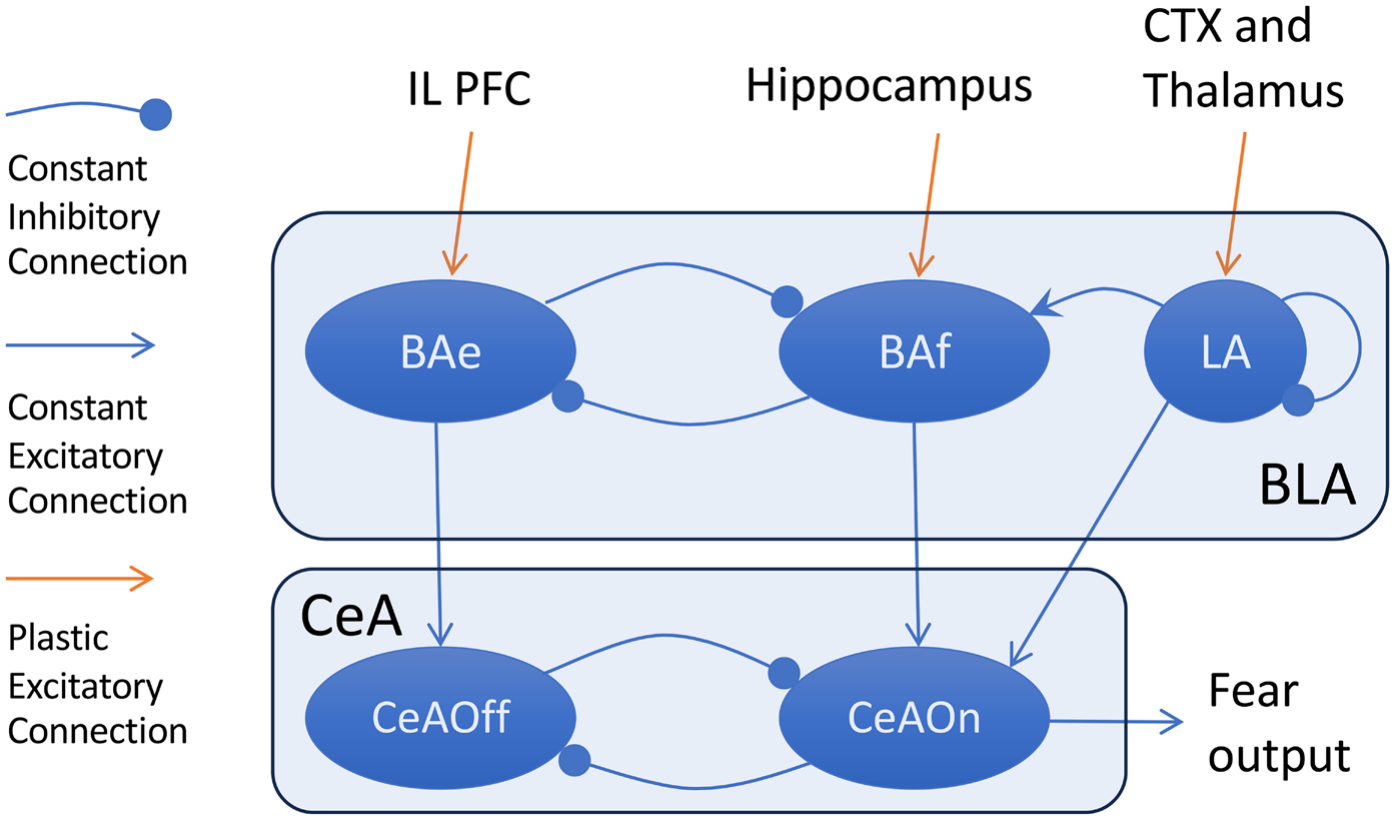
Diagram of the amygdala circuitry implemented in the model. LA, lateral amygdala, BAe and BAf are extinction and fear groups of the basal amygdala. CeAOff and CeAOn are pro‐extinction and pro‐fear neural groups in the central amygdala, respectively.

The basolateral amygdala (BLA) is a crucial component of the limbic system, playing a pivotal role in processing emotions like fear and anxiety (Janak & Tye, 2015). The BLA sends glutamatergic projections to the central amygdala (CeA) and other downstream targets. The BLA is comprised of LA and BA, which differ according to inputs and function. The LA, crucial to fear acquisition, receives thalamic and cortical inputs (Sun et al., 2020; Figure 1). The BA contains two functionally opposing groups of projection neurons: fear neurons (BAf), which are activated by aversive stimuli (Ciocchi et al., 2010), and extinction neurons (BAe), which inhibit fear responses (Herry et al., 2008; Figure 1). BAf neurons are essential for the expression of conditioned fear responses, receiving inputs from LA and the hippocampus (Herry et al., 2008; Moustafa et al., 2013; Pare & Duvarci, 2012), and exciting downstream pro‐fear neurons in the CeA (CeAOn) and other brain regions involved in fear behavior (Tovote et al., 2015). BAe neurons receive input from the infralimbic subdivision (IL) of the medial prefrontal cortex (mPFC) and are essential for extinction learning, increasing in activity as CS responses fade in the absence of the US, coupled with reduced BAf neuron activity (Herry et al., 2008). These shifts in neuronal activity are associated with reduced fear expression and increased CS approach behaviors (Milad & Quirk, 2012). Evidence suggests that BAf and BAe neural groups are mutually inhibiting (Figure 1) (Janak & Tye, 2015; Kim et al., 2016; Pare & Duvarci, 2012). The antagonistic relationship between these two BLA neuronal populations is essential for the appropriate regulation of emotional responses. This mutual inhibition must be mediated by interneurons since BA projection neurons are excitatory. While the contribution of these interneurons in gating BLA inputs is relatively well understood (Tovote et al., 2015), the exact mechanisms by which they might modulate mutual inhibition between fear and safety neurons remain ambiguous (Kim et al., 2016; Tully et al., 2007). This uncertainty underscores the need for further exploration, including the development of a computational model, to provide clarity. In our present model, we code the interaction between the BA groups of projection neurons as direct mutual inhibition (Figure 1). To justify this reduction, we simulated a BLA model with explicit representation of the interneurons and showed that it behaved very similarly to the reduced model used here (Kuznetsov, 2025). In short, interneuron dynamics are completely determined by the projection neurons' inputs and may only introduce a small delay into the dynamics, which is qualitatively negligible.

#### Central amygdala

The circuitry of the CeA is also quite complex, garnering extensive investigation. Like the BLA, the CeA is divided into pro‐fear (CeAOn) and pro‐extinction (CeAOff) neurons (Pare & Duvarci, 2012; Whittle et al., 2021). In contrast to the BLA, all CeA neurons are inhibitory and directly inhibit one another (Babaev et al., 2018), providing another layer of inhibition between the fear and extinction pathways (Figure 1). Medial (CeM) and lateral (CeL) CeA neurons are physiologically distinct: CeM receives denser projections from hypothalamic and brainstem regions involved in autonomic control, whereas CeL receives more cortical and sensory input (Bienkowski & Rinaman, 2013). However, both contain populations of neurons with overlapping functional properties in fear expression. Studies have demonstrated that pro‐fear neurons in both regions respond similarly to conditioned fear stimuli and are activated by shared upstream inputs like the BLA (Ciocchi et al., 2010; Haubensak et al., 2010), with LA neurons connecting with pro‐fear CeM neurons and BAf neurons projecting to the CeL. Based on these similarities, we group pro‐fear neurons from CeM and CeL as CeAOn. Conversely, BAe neurons project to pro‐extinction neurons in the CeA, CeAOff (Ciocchi et al., 2010). Therefore, BLA‐to‐CeA connections comprise mutually inhibiting pro‐fear and pro‐extinction pathways (Figure 1). We arrived at the reduced amygdala circuitry in Figure 1, acknowledging that additional components and pathways (Figure S1) can be added in the future. The pro‐fear CeM neurons project to downstream targets to induce fear‐related behaviors (Pare & Duvarci, 2012), with activation increasing heart rate, blood pressure, and freezing behavior, characteristic physiological responses to fear. Therefore, we consider CeAOn neurons the output of the model (Figure 1).

### Alcohol‐induced modulations

Alcohol modulates the weights of the connections between various subdivisions of the amygdala as well as its afferents. Acute alcohol increases inhibition in the BLA and CeA, elevating inhibitory gamma aminobutyric acid (GABA) transmission by ~50% (Munshi et al., 2023; Roberto et al., 2003; Zhu & Lovinger, 2006). Simultaneously, acute alcohol reduces excitatory glutamatergic transmission in the CeA by ~20% (Roberto, Madamba, et al., 2004). Accordingly, we modulate the synaptic weights to reflect these changes in the model (Table 2). Following chronic alcohol, the most prominent change observed is a fourfold increase in baseline GABA levels in the CeA (Roberto, Madamba, et al., 2004), with a much more modest increase in inhibitory postsynaptic current amplitude (~60%). Accordingly, we strengthen the inhibitory interaction in CeA by this lower amount (Table 3). Increased GABA levels also elevate the tonic inhibitory current in the CeA (Gilpin et al., 2015). Thus, we introduce negative drive to all CeA neurons (see the Methods section, Equations 4 and 5). There are reports of increased glutamate transmission in the BLA in the chronic alcohol condition (Läck et al., 2007), but in the CeA, reports are somewhat inconsistent (Roberto et al., 2012), except for the response to an alcohol challenge (Roberto, Schweitzer et al., 2004). To account for this BLA glutamate increase, we strengthen the input from LA to BAf neurons in the chronic alcohol condition (Table 3). Plasticity of excitatory inputs to the BLA from thalamus, hippocampus, and mPFC is defined by the conditioning protocol (see the Methods section). We will show that they undergo greater potentiation in the chronic alcohol condition (see the Results section).

We simulate the behavior of the resulting circuitry (Figure 1) to bridge the gaps between in vivo and in vitro techniques used to acquire the above data, linking neural activity changes with behavior. Specifically, we analyze how amygdala connectivity altered by alcohol could relate to altered behavioral responses during fear acquisition and extinction. We simulate two conditions: acute alcohol and prior repeated alcohol.

## METHODS

### Firing rates of neural groups

The model is implemented using standard firing rate formalism (Cowan et al., 2016). The rationale for this choice is, first, that it directly describes firing rates measured in experiments and, second, it links the data to large‐scale neural recordings most relevant to behavior. The equations for the activity of the BA Fear (BAf) and Extinction (BAe) neural groups (Figure 1) are ×the following:

(1) $$\frac{dU_{\mathrm{BAf}}}{\mathrm{dt}}=\frac{F\left(-W_{\text{BAinhib}}\times U_{\mathrm{BAe}}+W_{\mathrm{LA}\to F}\times U_{\mathrm{LA}}+W_{\mathrm{Hip}\to F}\times I_{\mathrm{Hip}}+{\mathrm{Dr}}_{\mathrm{BA}}\right)+\eta -U_{BAf}}{\tau }$$

(2) $$\frac{dU_{\mathrm{BAe}}}{\mathrm{dt}}=\frac{F\left(-W_{\text{BAinhib}}\times U_{\mathrm{BAf}}+W_{\mathrm{PFC}\to E}\times I_{\mathrm{PFC}}+{\mathrm{Dr}}_{\mathrm{BA}}\right)+\eta -U_{BAe}}{\tau }$$

Here, $U_{X}$ is the firing rate of neural group X, $W_{X\to Y}$ is the weight of the synaptic connection from group X to group Y. The signs in front of them define if these are excitatory (+) or inhibitory (−). τ is the time constant and ${\mathrm{Dr}}_{\mathrm{BA}}$ is the internal drive for BA neurons. $I_{\mathrm{Hip}}$ and $I_{\mathrm{PFC}}$ are firing rates of inputs that excite BAf and Bae, respectively. The η is normally distributed noise added for biological realism. F is the sigmoidal function:

$$F\left(x\right)=\frac{1}{1+e^{-\mathrm{Ex}\;\times \;\left(x-\mathrm{Th}\right)}}.$$

The parameters Ex and Th in this function represent neural excitability (or gain) and activation threshold, respectively. These parameters are redundant since they can be adjusted by scaling the synaptic weights and input drives introduced above. Therefore, we chose them to be the same for all neural groups to reduce the number of parameters to be calibrated (see the Calibration section).

We model a total of five neuronal populations using this formalism (Figure 1). The three remaining groups are modeled by the following equations:

(3) $$\frac{dU_{\mathrm{LA}}}{\mathrm{dt}}=\frac{F\left(-W_{\text{LAinhib}}\times U_{\mathrm{LA}}+W_{\text{Thal}\to \mathrm{LA}}\times I_{\mathrm{LA}}+{\mathrm{Dr}}_{\mathrm{LA}}\right)+\eta -U_{\mathrm{LA}}}{\tau }$$

(4) $$\frac{dU_{\mathrm{On}}}{\mathrm{dt}}=\frac{F\left(W_{\mathrm{BAf}\to \mathrm{CeA}}\times U_{\mathrm{BAf}}+W_{\mathrm{LA}\to \mathrm{CeA}}\times U_{\mathrm{LA}}-W_{\text{CeAinhib}}\times U_{\mathrm{Off}}+{\mathrm{Dr}}_{\mathrm{CeA}}\right)+\eta -U_{\mathrm{On}}}{\tau }$$

(5) $$\frac{dU_{\mathrm{Off}}}{\mathrm{dt}}=\frac{F\left(W_{\mathrm{BAe}\to \mathrm{CeA}}\times U_{\mathrm{BAe}}-W_{\text{CeAinhib}}\times U_{\mathrm{On}}+{\mathrm{Dr}}_{\mathrm{CeA}}\right)+\eta -U_{\mathrm{Off}}}{\tau }$$

The structure is similar to one in earlier modes (Carrere & Alexandre, 2015).

### Synaptic plasticity

As stated in the introduction, fear acquisition and extinction are linked to plasticity of synaptic connections within the amygdala and its inputs. Potentiation of synaptic inputs that transmit stimuli and context‐related information to the amygdala is key in modulating fear responsiveness (Pare & Duvarci, 2012; Ressler & Maren, 2019). Therefore, we assume plasticity of connection strength for all three inputs modeled (thalamus to LA, hippocampus to BAf, and mPFC to BAe). To focus on the contribution of these three inputs, all other connections are given constant synaptic weights. An important simplification achieved by this assumption is that task‐dependent plasticity and alcohol‐dependent plasticity affect different synapses in the model. This simplification decreases complexity due to mutual interdependence of plasticity processes. The plastic synaptic weights of the inputs to the LA ($W_{\text{Thal}\to \mathrm{LA}}$) and the BAf ($W_{\mathrm{Hip}\to F}$) implement a variation of the Rescorla–Wagner (Hebbian type) learning rule (Li & McNally, 2014):

(6) $$∆W_{\mathrm{Pre}\to \text{Post}}=\mathrm{ERR}\times \mathrm{US}\times \alpha \times I_{\mathrm{Pre}}\times U_{\text{Post}}$$

where $∆W_{\mathrm{Pre}\to \text{Post}}$ is a change in the synaptic weight, α is a plasticity rate constant, the US is the adverse unconditioned stimulus that engenders learning and the $I_{\mathrm{Pre}}$ and $U_{\text{Post}}$ are the respective firing rates of the pre‐ and postsynaptic neurons (the difference in notation is to emphasize that $I_{\mathrm{Pre}}$ is a parameter and $U_{\text{Post}}$ is a variable). The prediction error is calculated as:

(7) $$\mathrm{ERR}=\mathrm{US}-U_{\text{CeAOn}}.$$

using the accepted notation that an adverse unexpected stimulus is a positive prediction error, whereas a negative error is an unexpected omission of the stimulus (Moustafa et al., 2013).

For the synaptic weight projecting to BAe, the following was used:

(8) $$∆W_{\mathrm{PFC}\to E}=-\mathrm{ERR}\times \alpha \times I_{\mathrm{PFC}}\times U_{\mathrm{BAe}}.$$

In other words, inputs to the fear pathway are potentiated only in the presence of a shock (US = 1). In the absence of a shock (US = 0), inputs to the extinction pathway are potentiated until the prediction error vanishes as $U_{\text{CeAOn}}\to 0$. Using these rules, a negative prediction error leads to inhibition of the fear pathway not by a decrease in a synaptic weight, but by potentiation of an alternative pathway (Moustafa et al., 2013). This is justified by little evidence of synaptic depression in either fear acquisition or extinction. It is widely accepted that activating alternate pathways is key in fear extinction (Li & McNally, 2014).

### Behavioral protocol

**FIGURE 2:**
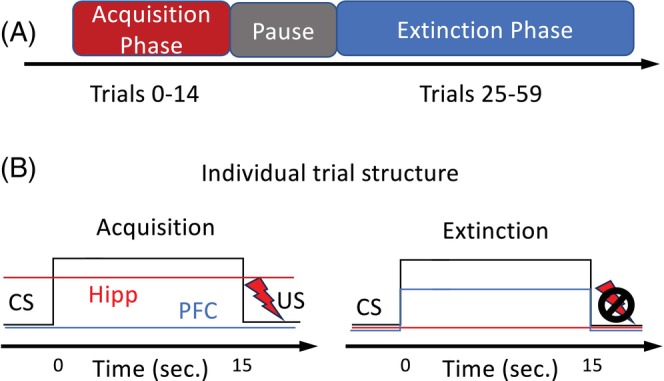
Design of the simulated trial structure. (A) A simulated session consists of 25 fear acquisition trials followed by 35 fear extinction trials. (B) Each individual trial is simulated for 15 sec. followed by an intertrial interval. For the acquisition trials (left), a CS represented by a thalamic input to the LA and context represented by a hippocampal input (Hipp) to the BAf are on. For the extinction trials (right), the CS input is active again, but a mPFC group projecting to BAe is on instead of the hippocampal input. Each acquisition trial ends with a shock (UC), whereas in the extinction trials, the shock is omitted.

Learning involves two phases: fear acquisition and fear extinction (Figure 2A). Acquisition consists of 15 trials, followed by 10 trials of “home cage rest,” where no stimulus is presented, and by extinction, the final 35 trials. Each acquisition trial starts with the presentation of context as a hippocampal input (Hipp) to the BAf and the CS in that context (Figure 2B, Acquisition) as a thalamic input to the LA. Contexts are represented as continuous signals spanning all acquisition trials, and the CS lasts for the 15‐sec trial duration (Moustafa et al., 2013; Vlachos et al., 2011). After 15 seconds, the network's prediction of an aversive US—that is, the activity of CeAOn—is read out, then compared with the actual US, which is 1 for acquisition and 0 for extinction trials. In extinction, no context signal is activated (Figure 2B, Extinction) to emphasize that the acquisition and extinction contexts differ, as in prior work (Carrere & Alexandre, 2015; Vlachos et al., 2011). Thus, the Hipp input to BAf is turned off in the model because the new context is not associated with fear. By contrast, an mPFC projection to BAe is activated during extinction (Carrere & Alexandre, 2015), and synaptic connections from mPFC to BAe strengthen according to the plasticity rules described above. Synaptic weights are updated after each trial based on the difference between the predicted and actual US. Initially, each trial was followed by an intertrial interval of 60 s or more to let the animal recover from the shock. However, we saw that, in the model, the firing rates equilibrate rapidly so we limited simulation of the intertrial interval to 7.5 s. This allows the system to fully reset for the next trial.

### Calibration

All activity levels were normalized to be between 0 and 1 for this model. To calibrate the model (Table 1), we combine functional criteria of fear acquisition and extinction with available electrophysiological data on synaptic currents and firing rates of each neural group. We use a current‐frequency relationship for CeA neurons (Haubensak et al., 2010) to calibrate the neural response function $F\left(x\right)$: at Ex=10; a unit of input in the model represents ~100 pA current. We generalize this scaling to all neurons in the model to compensate for sparse data. The chosen threshold, Th, of 0.5 ensures both low basal activity of all groups and ready responses when the inputs are activated. The internal drive for neural group X, ${\mathrm{Dr}}_{X}$, serves the same function as Th but allows for individual group modulation. Due to the redundancy of this parameter with inputs, the value of zero suffices for all neural groups in most conditions but is changed for CeA in the chronic alcohol case to account for greater inhibitory tone. For CeA neurons (including CeAOn and CeAOff), EPSP and IPSP amplitudes have been measured to be about 65 and 150 pA, respectively (Roberto et al., 2003; Roberto, Schweitzer et al., 2004). While these data are a good initial approximation for the model, they lack such information as convergence of the inputs, which affects the activation of these currents. To close this gap, we conducted parametric analysis, varying the parameters over a wide range and analyzing the changes in activity simulated by the model (see Figures S2 and S3). We observed wide parameter intervals where the dynamics of the model remain qualitatively the same. This gives us freedom to choose the parameter values. We assign a greater weight for the connection from BAe to CeAOff ($W_{\mathrm{BAe}\to \mathrm{CeA}}$) to robustly reproduce fear extinction (Table 1). We used the same connection weight for both LA and BAf inputs to CeAOn ($W_{\mathrm{BAf}\to \mathrm{CeA}}$ = $W_{\mathrm{LA}\to \mathrm{CeA}}$) because the data do not differentiate these neural groups (Roberto et al., 2003; Roberto, Schweitzer et al., 2004). IPSP amplitudes in LA neurons were up to 100 pA (Silberman et al., 2007; Vogel et al., 2016), so the inhibitory synaptic weight within LA was calibrated to be 1 in the model. The weight of LA‐to‐BAf connections determines the residual BAf response to CS after the animal changes context (e.g., extinction) (Pare & Duvarci, 2012). The strength of this connection was not measured directly, and thus we use this functional criterion. Lastly, our calibration of mutual inhibition between BAe and BAf (Woodruff & Sah, 2007) allows for coactivation of fear‐ and extinction‐responsive neurons in BA, as previously observed experimentally (Herry et al., 2008). Thus, we assign a much lower value of 0.13 for the inhibitory synaptic weight within BA.

**TABLE 1:** Model parameters. Values are calibrated as described in the Methods section.

Parameter name	Parameter description	Parameter value
$W_{\mathrm{LA}\to \mathrm{BAf}}$	Excitatory synaptic weight from LA to BAf	0.49
$W_{{\mathrm{BA}}_{\text{inhib}}}$	Inhibitory synaptic weight within BA	0.13
$W_{{\mathrm{LA}}_{\text{inhib}}}$	Inhibitory synaptic weight within LA	1.0
$W_{\mathrm{BAf}\to \mathrm{CeA}}$	Excitatory synaptic weight from BAf to CeAOn.	0.65
$W_{\mathrm{LA}\to \mathrm{CeA}}$	Excitatory synaptic weight from LA to CeAOn.	0.65
$W_{\mathrm{BAe}\to \mathrm{CeA}}$	Excitatory synaptic weight from BAe to CeAOff.	0.98
$W_{\mathrm{Ce}A_{\text{inhib}}}$	Inhibitory synaptic weight within CeA	1.5
τ	Time constant for neural activities	0.05
α	Learning rate constant	1

The time constant τ is set to 0.05 s, accounting for delays in responses of neural groups. We tested our results across a wide parameter range and found little impact on simulated trial duration since it is significantly greater than the time constant. This requirement is met for typical trial duration of 15 s and was used in previous modeling (Carrere & Alexandre, 2015).

### Stability and simulations

Our model was coded and all results were generated in Python. We used Euler's method, which simply increments each variable by the value given by the Equations (1–5) scaled by the time step (Griffiths & Higham, 2010). This is sufficient for solving this system given its strong convergence to steady states. A step size of 15 ms is used, and each trial described above consists of 1000 timesteps. This brings the simulated trial duration to 15 s (Figure 2B), which is typical for experimental design. Each trial is followed by a 7.5‐s intertrial interval, adding another 500 steps. We tested other durations for both trial and intertrial intervals (5–60 s) and timesteps (2–30 ms) and ensured that our results remain stable in a very wide range of these parameters.

All nuclei include additive normally distributed noise with a mean of 0.03 and an amplitude of 0.1, which is 10% of the maximal neural activity level. This noise ensured the stability of the simulations and model dynamics to random perturbations. To quantify the influence of this noise on the dynamics, we simulated 100 model instances and averaged the number of trials needed for fear acquisition and extinction over the model instances. We calculated the standard deviations of these trial numbers, provided in the text below. The stability of the results with respect to the influence of noise was combined with that with respect to parameter changes and showed wide ranges of parameters where the dynamics of the model remain qualitatively the same (see Figures S2 and S3).

## RESULTS

### Fear acquisition

The first 15 trials of the simulations tested fear acquisition. During these trials, the model network receives a CS input activating the LA and a context input activating the BAf. The CS inputs are maintained until the presentation of a US (shock), and the context input is kept active for the duration of the session. The US is represented in the calculation of the prediction error (see the Methods section).

**FIGURE 3:**
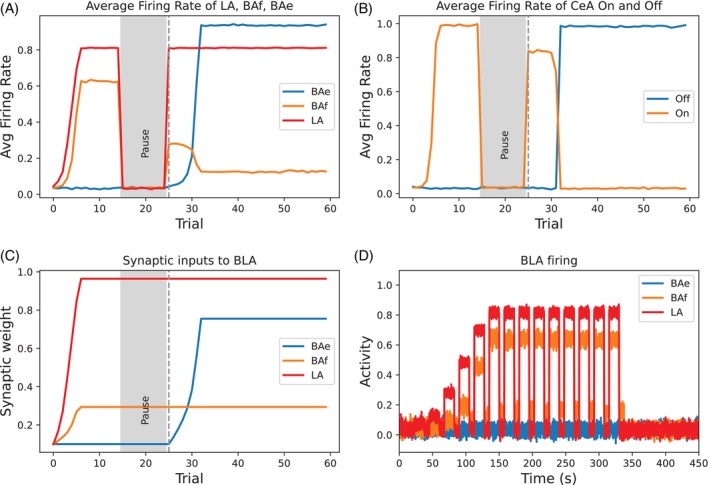
Dynamics of amygdala activity and synaptic inputs during fear acquisition and extinction. Acquisition trials are 0–14. Extinction trials are 25–59. Trials 15–24 represent a pause or “home cage rest” (shaded). The vertical dashed line marks the first trial after the pause representing fear recall in a new context. (A) Average activity levels of LA, BAf, and BAe. (B) Average activity levels of CeAOn and CeAOff. (C) Weights of synaptic inputs to LA, BAf, and BAe. (D) Exemplary time dependence of the activity levels throughout the first 20 trials for the BLA neural groups. The averaging in (A) and (B) is done over the first 15 sec of each trial, whereas the remaining 7.5 s is an intertrial interval.

**FIGURE 4:**
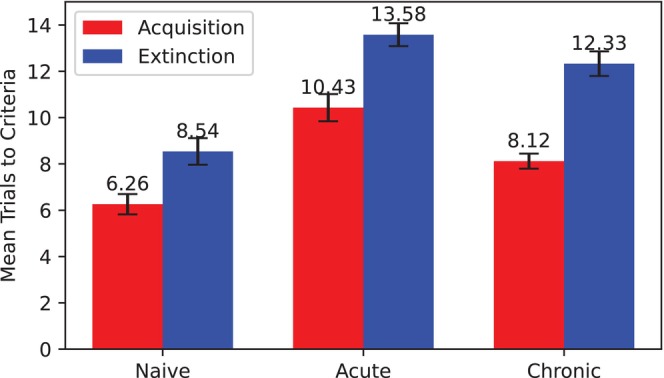
Number of trials to criteria (i.e., fear response in acquisition and the absence of it in extinction) for the control (Naïve), acute and chronic alcohol conditions. The bars show the mean, and the brackets show the standard deviation over 100 sessions.

Figure 3 shows trial‐by‐trial dynamics of the averaged activity of all amygdala neuron types modeled (A,B), and plastic synaptic weights (C). The last panel (D) shows exemplar neural activity within the first several trials. Acquisition trials (0–14) are to the left of the shaded area. The activity of each neural group is averaged for the duration of each trial. As the figure shows, the activity of all neural groups is low at the beginning of the acquisition process. The initial growth of activity is very slow, followed by a sudden increase in the activation of LA, BAf, and CeAOn groups. Acquisition takes on average 6.26 trials (SD 0.44; Figure 4), after which activity remains at approximately the same level. The behavioral readout of the model is the activation of the CeAOn neurons, generating a fear response to the CS. This completes the fear acquisition process.

The mechanism for fear acquisition in the model is potentiation of synaptic weights from the sensory areas and hippocampus to LA and BAf, respectively. Their dynamics are shown in Figure 3C. Potentiation of these two projections is due to a positive prediction error: the difference between the punishing US presented at the end of the trial and the lack of CeAOn activation. However, the initial growth of the synaptic weights is very gradual, due to the Hebbian plasticity rule (see the Methods section), which makes the changes in synaptic weights dependent on the activity levels of both pre‐ and postsynaptic neurons. Thus, the low initial activity of LA and BAf impedes fast potentiation of the synapses. The prediction following from this mechanism is that fear acquisition can be accelerated by directly priming the activity of the LA and/or the BAf neurons.

Trials 15–24 model a pause in fear conditioning, during which no stimulus is presented to the model. No changes in the synaptic weights occur during this period (Figure 3C, shaded area), whereas the fear output is absent (Figure 3B) because no CS is presented, and the agent is not receiving an input corresponding to the context associated with the US. Trial 25 (Figure 3 dashed vertical line) plays the role of fear recall and shows activation of the LA at the level achieved during acquisition. A much lower BAf activation is due to a shift to a different context, which is modeled by no activation of hippocampal projections to BAf. Indeed, a set of hippocampal neurons responsive to the new context is not associated with fear and, thus, does not activate the BAf. Therefore, the model replicated fear recall after a pause in learning.

### Robust fear extinction

Fear extinction was tested beginning with the fear recall in trial 25 until the end of the simulation. During these trials, the CS continues to activate the LA as it did during the acquisition trials. The context, however, changes, as mentioned above. This produces a lower activation of the BAf group. Furthermore, as extinction develops, projections from a set of IL neurons responsive to this context potentiate and activate the BAe (Herry et al., 2008). In the model, therefore, we silence the input from the hippocampus to the BAf and activate mPFC neurons that provide weak initial stimulation of the BAe. No US is presented during extinction trials, which is incongruent with the persistence of the fear response.

Figure 3 shows that at the beginning of extinction, BAf activation was much weaker than the level achieved in acquisition (Panel A, compare trials 14 and 25) because BAf was not excited by the hippocampus due to the change in context. On the other hand, there is a very modest drop in the activation of CeAOn (Figure 3B) due to persistent excitation from the LA (Figure 3A). Therefore, the model reproduces initial learned fear CS responses regardless of the context, with persistent activity of the LA reflecting its direct activation by the CS.

The immediate drop induced by the context change is followed by a gradual further decrease in the activity of BAf and CeAOn, as well as a gradual increase in the activity of BAe. This continues for 7 trials, after which there is a sharp increase in BAe and CeAOff activity. Simultaneously, the activity of BAf and CeAOn sharply decreases. After this occurs in trial 33, there is little change in the activities of any of the system's neural groups. LA activity remains constant throughout this process, reflecting the same CS presentation in all trials. As the activity of CeAOn remains near zero, we register fear extinction. On average, it takes 8.54 trials (SD 0.57, Figure 4).

The mechanism for extinction is potentiating the synaptic weight between the mPFC and the BAe. Figure 3C shows the slow increase in this weight following trial 25: gradual at first due to the low activity of BAe, the postsynaptic neural population, then increasing potentiation as the activity levels of BAe increase. It is important to note that extinction does not occur by depression of any synaptic pathways potentiated during acquisition (Figure 3C red and orange). Rather, it occurs by strengthening an alternative pathway, which inhibits fear‐responsive neural groups within the amygdala circuitry. Thus, mutual inhibition within the amygdala determines the capacity for extinction.

### Acute alcohol impedes fear extinction by differentially affecting BLA and CeA activity

**FIGURE 5:**
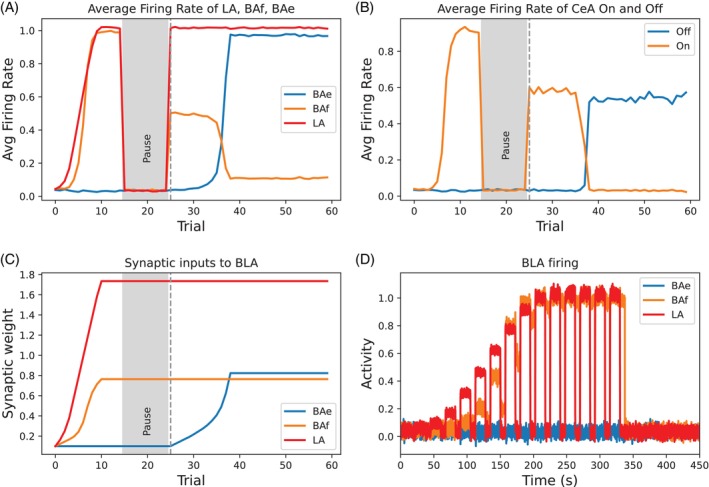
Effects of acute alcohol on amygdala activity during fear acquisition and extinction. Notation is the same throughout the panels as in Figure 3. Parameter values changed from control are in Table 2.

As mentioned in Methods, acute alcohol weakens the excitatory inputs to CeA and strengthens inhibition within both LA and CeA. While the direction of the changes is clear, the magnitude is hard to calibrate due to insufficient experimental data. In particular, aligning changes in the amygdala recorded in vivo with changes in synaptic function, measured as transmitter release or receptor activation in vitro, presents a challenge. To close this gap, we refer again to our parametric analysis (see Figures S2 and S3). The analysis shows that the above parameter modulations exert robust activity changes over a wide range of physiologically relevant values. Figure 5 depicts the results obtained when parameters modulated by acute alcohol were set to the values in Table 2.

**TABLE 2:** Parameter modulation by acute alcohol. All other parameters stay the same.

Parameter	Modulation	Literature
$W_{{\mathrm{BA}}_{\text{inhib}}}=0.25$	Increased by ~50%	(Roberto et al., 2003; Silberman et al., 2007; Weiner & Valenzuela, 2006; Zhu & Lovinger, 2006)
$W_{{\mathrm{LA}}_{\text{inhib}}}=1.5$	Increased by ~50%	(Roberto et al., 2003; Silberman et al., 2007; Weiner & Valenzuela, 2006; Zhu & Lovinger, 2006)
$W_{\mathrm{BAf}-\mathrm{CeA}}=0.4$	Decreased by ~20%	(Roberto, Madamba, et al., 2004)
$W_{\mathrm{BAe}-\mathrm{CeA}}=0.6$	Decreased by ~20%	(Roberto, Madamba, et al., 2004)
$W_{\mathrm{Ce}A_{\text{inhib}}}=2.5$	Increased by ~50%	(Roberto et al., 2003; Silberman et al., 2007; Weiner & Valenzuela, 2006; Zhu & Lovinger, 2006)

Figure 4 indicates that acquisition in the presence of alcohol is achieved after an average of 10.43 trials (SD 0.59), which is 3 trials longer than in the control condition. This is directly caused by a reduction in the BLA‐to‐CeA connection strength. Since, as before, conditioning is contingent on sufficient excitation of the CeAOn neurons, stronger excitation of their inputs is required to compensate for the reduced synaptic weight. Thus, greater activation of LA and BAf (Figure 5A) results from greater potentiation of the corresponding connections (Figure 5C). Achieving this additional potentiation requires a greater number of trials. Furthermore, LA activation reaches its maximum, so further activation of BAf neurons is required for the additional increase in input to CeAOn (Figure 5A vs.  3A). Therefore, our simulations predict that acute alcohol increases the contribution of BAf to fear conditioning.

Extinction takes far longer in the acute alcohol condition, increasing from 8.54 trials (control) to an average of 13.58 trials (SD 0.49; alcohol) (Figure 5, trials 25–38). Thus, the model reproduces delayed extinction under acute alcohol (Bisby et al., 2015; Lattal, 2007), largely as a consequence of the stronger activation of LA and BAf during fear acquisition. This stronger activation of the fear pathway constitutes a larger barrier to overcome to inhibit the fear response. Not only must BAe activity now reach a greater level to achieve extinction, but its activation is further impeded by inhibition from BAf neurons. This slows down potentiation of the BAe input throughout extinction since the potentiation rate explicitly depends on BAe activation (see the Methods section). Thus, increased inhibition between BAf and BAe under acute alcohol further impedes fear extinction via the same mechanism: stronger inhibition amplifies the influence of the BAf fear response over BAe activity, preventing growth of BAe activity.

Our simulations show that the increased inhibition in the CeA during acute alcohol exposure does not significantly affect behavior during conditioning but contributes to the overall reduction of CeA activation levels (Figure 5B).

Overall, the model predicts that fear acquisition and extinction under acute alcohol generate greater activation of the corresponding BLA neural groups but lower CeA activity. Importantly, the activity of the CeAOff neurons after fear extinction is reduced most (~40%) by acute alcohol, compared with control conditions (Figure 5B blue).

### Chronic alcohol reduces reliability of fear extinction by inhibiting CeA activity

**FIGURE 6:**
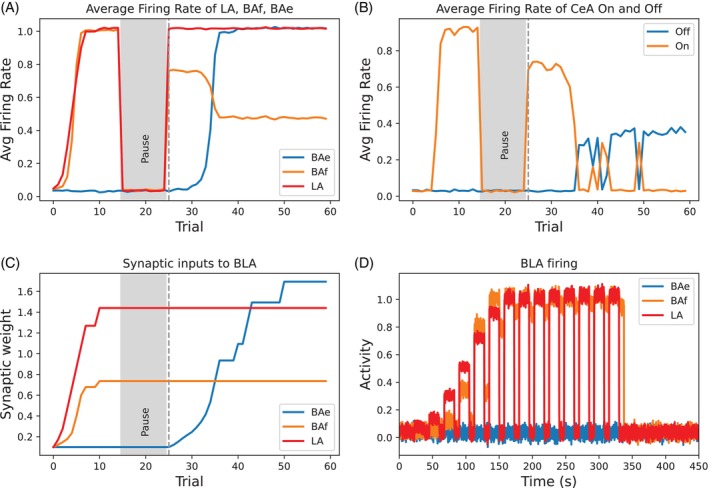
Effects of chronic alcohol on amygdala activity during fear acquisition and extinction. The notation is the same as in Figure 3. Parameter values changed from the control are in Table 3.

Prior repeated alcohol exposure increases inhibition in the CeA and excitation of BAf by LA (see the Methods section and Table 3). The number of trials to acquire fear is slightly greater after repeated alcohol exposure (Figure 4; 8.12 vs. 6.26; SD 0.32). However, activation of the fear pathway is substantially changed: LA activation achieves a higher maximum value and BAf activation is greatly increased (Figure 6A). These changes are like those produced by acute alcohol, but repeated alcohol increases the input to the CeAOn neurons as CeA synaptic weights do not decrease in chronic alcohol. Higher CeAOn excitation is required for fear conditioning following chronic alcohol because it counteracts the amplified tonic inhibition in CeA. Thus, the modulations of the amygdala produced by chronic alcohol, however different from acute alcohol, produce very similar changes in the activity levels (Figures 5A,B and 6A,B).

**TABLE 3:** Parameter modulation by chronic alcohol. All other parameters stay the same.

Parameter	Modulation	Literature
$W_{\mathrm{Ce}A_{\text{inhib}}}=2.5$	Increased by ~60%	(Roberto, Madamba, et al., 2004)
${\mathrm{Dr}}_{\mathrm{CeA}}=-0.5$	Increased tonic GABA levels (4‐fold) and IPSC frequency (0.22–0.72 Hz)	(Gilpin et al., 2015)
$W_{\mathrm{LA}-\mathrm{BAf}}=0.6$	Increased by ~20%	(Läck et al., 2007)

Fear extinction trials start with substantially greater residual activation of both LA and BAf (Figure 6A trial 25). Thus, extinction is achieved later than in control conditions, requiring on average 12.33 trials (SD 0.53). Our simulations reproduce impeded fear extinction after chronic alcohol (Bertotto et al., 2006; Holmes et al., 2012; Smiley et al., 2020). Furthermore, the CeAOff activation levels after extinction, although sufficient to suppress the fear response, remain low. This parallels findings for acute alcohol, but the effect is amplified: CeAOff activity after extinction is down an additional 20% compared with acute alcohol (Figure 4B vs. 5B, blue). This activation level is so low that noise added to our simulations can switch the CeA and induce a fear response (Figure 6B trial 47). In the simulation, the system has the capacity to further strengthen the extinction pathway (Figure 6C), but CeAOn activation remains low (Figure 6B trials 48–60), making fear extinction much less reliable after chronic alcohol due to inhibited CeA neuron activity.

### Robustness analysis for fear extinction

The functional robustness of fear extinction is determined by how strongly activation of the extinction pathway, BAe and CeAOff, inhibits CeAOn (Figure 1). Thus, two factors are contributing: first, how much IL inputs can activate the extinction pathway, and second, how strong is mutual inhibition in BA and CeA. Roughly, extinction is achieved if the sum of the inhibitory and excitatory inputs to CeAOn (Equation 4) is below the threshold of 0.5 for the response function $F\left(x\right)$:

$$W_{\mathrm{BAf}\to \mathrm{CeA}}\times U_{\mathrm{BAf}}+W_{\mathrm{LA}\to \mathrm{CeA}}\times U_{\mathrm{LA}}-W_{\mathrm{Ce}A_{\text{inhib}}}\times U_{\mathrm{Off}}+{\mathrm{Dr}}_{\mathrm{CeA}}<0.5$$

Here, the positive terms work to violate the inequality and the negative terms counteract them and work to satisfy the inequality, thus achieving extinction. Therefore, extinction occurs if:

$$W_{\mathrm{Ce}A_{\text{inhib}}}>\frac{W_{\mathrm{BAf}\to \mathrm{CeA}}\times U_{\mathrm{BAf}}+W_{\mathrm{LA}\to \mathrm{CeA}}\times U_{\mathrm{LA}}+{\mathrm{Dr}}_{\mathrm{CeA}}-0.5}{U_{\mathrm{Off}}}$$

**FIGURE 7:**
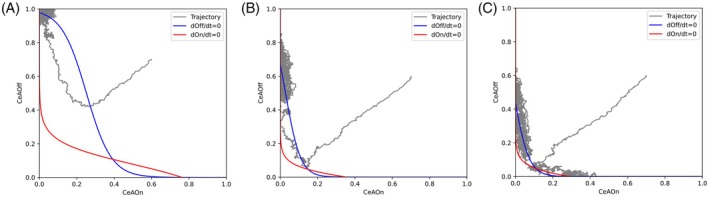
Within‐trial analysis of model dynamics in naïve (A), acute (B) and chronic (C) alcohol conditions. The system is reduced to two variables: Activation of CeAOn and CeAOff neurons. The red and blue curves are the nullclines of the corresponding equations. Their intersections are equilibrium states. The gray is a noisy trajectory showing robust activation of the CeAOff neurons in the naïve state (A), but their much weaker and less robust activation in acute (B) and especially in the chronic alcohol case (C).

In naïve conditions (Table 1) $W_{\mathrm{Ce}A_{\text{inhib}}}=1.5$, and, if we assume full activation of LA, BAf and CeAOff $\text{(}U_{\mathrm{BAf}}=U_{\mathrm{LA}}=U_{\mathrm{Off}}=1\text{)}$, the right‐hand side of this inequality is 0.8. This ensures robust fear extinction in naïve conditions and gives a range of parameters where it holds. The major effect, however, is not intuitive since it is mediated by a strong nonlinear decrease in CeAOff activity as lateral inhibition is strengthened (greater $W_{\mathrm{Ce}A_{\text{inhib}}}$, $W_{{\mathrm{BA}}_{\text{inhib}}}$, or lower ${\mathrm{Dr}}_{\mathrm{CeA}}$) or excitatory pathways are weakened (lower $W_{\mathrm{BAe}\to \mathrm{CeA}}$). To illustrate this decrease, we reduced the circuit to mutually inhibiting CeAOn and CeAOff groups (see Appendix S1 for reduction and explanation of the following analysis). The values of their BLA inputs are assumed to be equal to approximate levels reached in extinction for our simulations of naïve, acute, and chronic alcohol conditions (Figures 3, 4, 5; e.g., in the naïve case $U_{\mathrm{BAf}}=0.15,U_{\mathrm{BAe}}=0.9,U_{\mathrm{LA}}=0.8$). Phase plane analysis for this system is presented in Figure 7. Blue and red curves show nullclines of the two equations defined by $\frac{{\mathrm{dU}}_{\mathrm{Off}}}{\mathrm{dt}}=0$ and $\frac{{\mathrm{dU}}_{\mathrm{On}}}{\mathrm{dt}}=0$. Their intersections are equilibrium states of the system. An exemplar noisy within‐trial trajectory is shown in gray. It settles in the vicinity of one of the equilibria. Activation of the CeAOff group (vertical axis) at this equilibrium is maximal in naïve conditions (Figure 7A) and diminished in acute (B) and chronic (C) alcohol conditions. Noise, which is taken the same in all three cases, perturbs the trajectory much more in the alcohol conditions (Figure 7B,C). Furthermore, under chronic alcohol, the fear extinction capacity of the system has reached its maximum since activation of BAe is maximal (Figure 6A orange), and yet extinction is not robust (Figures 6C and 7C). Altogether, the model shows a mechanism for diminished fear extinction robustness due to strengthening of lateral inhibition and weakening of excitatory signal transduction in amygdala by acute and chronic alcohol.

## DISCUSSION

### Acute and chronic alcohol delay fear extinction

Acute (Bisby et al., 2015; Dickerson & Ferraro, 1976; Gulick & Gould, 2007; Kitaichi et al., 1995; Williams & Lattal, 2019) and chronic (Bertotto et al., 2006; Holmes et al., 2012; Stephens et al., 2005) alcohol have variable effects on fear acquisition. In our simulations, alcohol significantly increases the number of trials required for acquisition in both acute and chronic conditions (10.43 and 8.12, respectively, vs. 6.26), replicating several studies (Bisby et al., 2015; Dickerson & Ferraro, 1976; Stephens et al., 2005; Williams & Lattal, 2019). Our model suggests that inconsistency in the acquisition time may come from variable background activity in the BLA or its inputs before learning. The learning process involves Hebbian synaptic plasticity, primed by the activity of both pre‐ and postsynaptic neurons (Li & McNally, 2014). Greater initial activity speeds up fear acquisition, whereas lower initial activity delays acquisition. Such priming can be achieved optogenetically or by drugs like alcohol. If alcohol increased activity levels prior to learning, the acquisition time would be decreased relative to the values observed in control conditions. If, for example, hippocampal activity is reduced by acute alcohol (Melia et al., 1996), this should further slow context‐dependent learning, thereby increasing time to acquire extinction. In our simulations, we assume the same initial activity levels in all conditions to avoid variations in the acquisition time. However, our modeling suggests that fear acquisition time may be highly variable, depending on both background activity in the BLA and its input structures and the specifics of the task.

Conversely, fear extinction is delayed more consistently by both acute (Bisby et al., 2015; Lattal, 2007) and chronic (Bertotto et al., 2006; Holmes et al., 2012; Smiley et al., 2020) alcohol. Our model successfully reproduces this (13.58, 12.33, and 8.54 trials to extinction, respectively, in acute and chronic alcohol and control). What mechanisms control the onset of fear extinction? The simulations show that the number of trials required for fear extinction is largely independent of that required for conditioning. For example, the predicted variability in fear acquisition timing will not affect extinction. By contrast, the final levels of activity in the fear pathway after acquisition do affect extinction. Following both acute and chronic alcohol, LA and BAf are activated more strongly than in control conditions; thus, BAe receives stronger inhibition from BAf at the beginning of extinction. Accordingly, the longest extinction delay was obtained in the acute alcohol simulations, where BAf activity was nearly twice as high as the control case (Figure 6A vs. 3A, orange), and BA inhibition also was increased. The extinction delay is affected by the background firing rate of the mPFC input. Indeed, by the same Hebbian rule, this extinction delay depends on the background firing rate of postsynaptic BAe and presynaptic IL neurons. In our simulations, we assume the same mPFC activity levels in all conditions to focus on the influence of the amygdala structure on fear conditioning. Therefore, the delay in fear extinction in alcohol is mostly induced by greater BLA activation after fear acquisition via alcohol‐induced modulation of synaptic weights.

### Fear extinction is less robust in acute and chronic alcohol

In our simulations, both acute and prior repeated alcohol lead to greater activation of the BLA fear pathway following acquisition. In extinction, greater activation of BAe neurons also is required, but this does not lead to a greater activation of CeA neurons. On the contrary, all CeA neural groups are less active in alcohol. Indeed, greater BLA activation is a compensatory mechanism to counteract elevated inhibition and/or lower excitation in the CeA. The decrease in CeA activity during extinction is most significant for the CeAOff neurons under chronic alcohol (Figure 6B, blue), where activity is ~60% lower than control conditions (Figure 3B, blue). As a result, activation of the extinction pathway through CeA is insufficient to reliably oppose fluctuations in the fear pathway needed to exclude fear responses. Thus, the model predicts that acute and especially prior repeated alcohol shifts the amygdala network away from the robust extinction regime.

### Alcohol exposure changes context and stimulus contributions to fear conditioning

The aim of this study was to model the mechanistic relationships between amygdala function and structural adaptation in fear learning and the impact of acute and repeated alcohol on this process. In the model, LA and BAf projections to CeA mediate fear conditioning and may contribute differently depending on experimental conditions. BAf is activated more than LA in fear acquisition simulations under acute and chronic alcohol, whereas the LA contribution is greater in control conditions. The amplified BAf activity is caused by potentiation of hippocampal inputs and, thus, enhances contextual contributions to the fear memory. Our model predicts that fear memories formed in acute or after chronic alcohol are more strongly tied to the context.

The contributions of conditioned stimuli and context to fear acquisition can be manipulated by priming activity in one of the regions that provide their excitation: for example, thalamic areas for LA and the hippocampus for BAf. In particular, acute alcohol can drastically decrease hippocampal activity (Melia et al., 1996), which would compensate for greater potentiation of hippocampal input to BAf neurons. The current model does not account for these modulations, but rather provides a framework for future expansion of the model's predictive function. The model predicts that attempting to rescue contextual learning by restoring hippocampal activity in alcohol conditions will exaggerate the contextual contribution to fear acquisition.

### Comorbidity of alcohol use and PTSD

Our model suggests that alcohol impairs fear extinction, a hallmark of PTSD (Milad et al., 2009; Zuj et al., 2016). Comorbidity of PTSD and AUD is well documented (Petrakis & Simpson, 2017). We speculate that similar plasticity of amygdala synapses may occur in PTSD and AUD. Specifically, an experimental hypothesis to be tested is whether CeA excitation by BLA is lowered or internal CeA inhibition is elevated in PTSD. Alternatively, other mechanisms that weaken the extinction pathway may be affected in PTSD. Therefore, our model offers a mechanism that may explain the comorbidity of AUD and PTSD.

Additionally, PTSD likely involves a bias toward fear responses, not only a dysfunction of fear extinction. This bias could stem from several additional factors. First, fear memories are strengthened in PTSD (Rubin et al., 2008). Repeated traumatic experiences can persistently alter the BLA and connected areas. These changes may increase fear circuit sensitivity, making individuals with PTSD prone to over‐respond to neutral or ambiguous stimuli. Second, structures like the hippocampus and mPFC modulate BLA activity and often are found to be hypoactive in PTSD (Shin et al., 2004). This dysregulation can diminish control over fear circuits within the BLA, enhancing the fear bias observed in PTSD. These mechanisms are affected by alcohol use and exacerbate the PTSD phenotype. Importantly, females are more susceptible to stress effects on alcohol misuse and PTSD diagnosis, a possible future expansion of the model (see expanded discussion in Appendix S1).

### The model as a framework for elucidating the impact of modulators

A central purpose for developing this model is to generate a framework for examining the impact of various modulatory influences on fear learning. A topic related to such modulations is sex differences (see Appendix S1 for additional discussion). Here, we limited the factors incorporated to first build a basic model (see Appendix S1 for comparison with earlier models). By reproducing core fear conditioning experiments and the influence of alcohol, we show that the circuit represents core components necessary for these phenomena. There are multiple neuromodulators and neuropeptides implicated in amygdala plasticity following acute and chronic alcohol exposure (Roberto et al., 2012). Additionally, the complexity of the inputs modulating not only BA activity but also the interconnected amygdala subdivisions is far greater than accounted for in the current model design. Due to rigorous reduction techniques exercised while designing the model circuitry, it can be easily expanded to account for more complex structure. The addition of other amygdala inputs, like the intercalated cells or the prelimbic subdivision of the mPFC, would enable understanding of their interplay with the core modeled here. Thus, the model provides a scaffold for rapid assessment of modulators' efficacy to reduce fear behavior, particularly in the presence of alcohol, thereby identifying novel targets with the highest potential for further preclinical investigation. This could streamline the search for improved treatment targets to ameliorate comorbid stress‐ and alcohol‐related mental illnesses.

Our model demonstrates how plasticity within amygdala circuitry contributes to alcohol's effects on fear conditioning and makes the following predictions. First, the delay in fear extinction caused by alcohol is mostly induced by greater activation of the BLA after fear acquisition due to alcohol‐induced modulation of synaptic weights. Second, the model predicts that both acute and prior repeated alcohol shift the amygdala network away from a robust extinction regime by inhibiting activity in the CeA. Third, our model predicts that fear memories formed in acute or after chronic alcohol are more connected to the context, although this prediction is subject to variations in hippocampal activity by alcohol. Finally, our modeling suggests that fear acquisition time may be highly variable and dependent on the specifics of the task, which explains variable results on the influence of alcohol on this time.

## AUTHOR CONTRIBUTIONS

AL coded and calibrated the model, performed simulations, wrote the text, and prepared the manuscript. MLL contributed to the interpretation of the results and the writing and revising of the manuscript. AK led the design of the model and simulations, their interpretation, and edited the manuscript.

## FUNDING INFORMATION

The initial part of the work was funded by the NSF REU program DMS‐1559745. A. Kuznetsov acknowledges support from grants P60 AA007611 and U24 AA029970. M. Logrip acknowledges support from grant P60 AA007611.

## CONFLICT OF INTEREST STATEMENT

The authors declare that the research was conducted in the absence of any commercial or financial relationships that could be construed as a potential conflict of interest.

## Supporting information


Appendix S1.

## Data Availability

Data sharing not applicable to this article as no datasets were generated or analysed during the current study.
