# Supplementary material for: Mechanisms of alcohol influence on fear conditioning: A computational model
Source: Alcohol Clin Exp Res (Hoboken). 2025 May 19;49(6):1233–47. doi: 10.1111/acer.70071 (PMC12173769; doi:10.1111/acer.70071)
Supplement: Supplementary file 1 — Appendix S1. [file ACER-49-1233-s001.docx]

**Supporting information**

**Figure S1:** Amygdala circuitry including additional neural groups that mediate its signal transduction. In particular, two groups of BLA interneurons (I) are shown to mediate mutual inhibition between BAe and BAf neurons. In our model (Fig. 1), the interneuron groups are omitted as data about their properties are inconsistent. The two groups of central amygdala neurons representing fear state, CeLOn and CeMOn, are merged into one, CeAOn, due to their functional similarity. Additionally, two clusters of intercalated (ITC) neurons, dorsolateral (ITCd) and ventrolateral (ITCv), mediate the BLA to CeA pathway (Ciocchi et al., 2010). We have omitted them in this model as they repeat the connections that the model already accounts for. Specifically, ITCd neurons connect LA to CeAOff neurons (Ciocchi et al., 2010), providing inhibition of CeAOff. In a similar way, the LA inhibits CeAOff via CeAOn in the model. The ITCv neurons are excited by BA extinction neurons and provide inhibition of CeAOn neurons (Ciocchi et al., 2010). In a similar way, BAf neurons reduce CeAOn excitation via inhibition by CeAOff neurons in the model. Thus, the ITC pathways reinforce the mutual inhibition between fear and extinction pathways, which is accounted for by the model. In some circumstances, the two additional pathways may act differentially from those to which they are functionally similar, but this requires their differential inputs. Therefore, we merge the similar pathways and augment the strength of the LA to CeAOff and BAf to CeAOn bisynaptic connections to account for the model reduction.

**Parametric analysis of the model**

**Figure S2:** The model shows robust fear acquisition in a wide range of parameters. Fear acquisition is achieved in the grayed areas as the activation of the CeAOn neurons exceeds the threshold of 0.9 (green dotted line). Dependencies of mean CeA On (red) and Off (blue) neuron activation on the parameters averaged across multiple simulations is shown together with their standard deviations. The input parameters were fixed as in the fear acquisition simulations (CS=1.5, Hipp=0.5, IL=0) and the plastic synaptic weights in the fear pathway were set at the maximum allowed strength (BAf_input = LA_input = 2), whereas the BAe input synaptic weight was zero (BAe_input=0). Note that this choice results in the maximal activation of the fear pathway (U_LA = U_BAf = 1). Obviously, the model output does not depend on the parameters of the extinction pathway and inhibition between the pathways because during fear acquisition the extinction pathway stays inactive (U_BAe = U_Off =0). However, the output of the model depends on 2 parameters: CeAOn input weight and drive for the CeA neurons Dr_CeA. As these parameters decrease, activation of CeAOn neurons drops and, thus, dear conditioning becomes impossible. The graphs show that our choice of parameters for the naïve conditions (vertical dashed lines) is well within the range ensuring robust fear acquisition.

**Figure S3:** The model shows robust fear extinction in a wide range of parameters. Fear extinction is achieved in the grayed areas as the activation of the CeAOn neurons drops below the threshold of 0.1 (green dotted line). Dependencies of mean CeA On (red) and Off (blue) neuron activation on the parameters averaged across multiple simulations is shown together with their standard deviations. The input parameters were fixed as in the fear extinction simulations (CS=1.5, Hipp=0, IL=1) and the plastic synaptic weights in both the fear and extinction pathways were set at the maximum allowed strength (BAf_input = LA_input = BAe_input=2). In the explored parameter ranges, the model output does not depend on the parameters of the inhibition strength within BLA (BA_inhib and LA_inhib), nor on the strength of the LA to BAf activation connection (LA_to_BAf). As expected from the topology of the circuit, extinction holds for lower CeAOn input weight and greater CeAOff input weight and fails otherwise. Extinction also fails with diminishing CeA inhibition strength (CeA_inhib), whereas a decreasing drive for the CeA neurons (Dr_CeA) reduces activation of the CeAOff neurons, which created the loss of robust extinction (see main text). The graphs show that our choice of parameters for the naïve conditions (vertical dashed lines) is well within the range ensuring robust fear extinction.

**Reduction of the model for phase space analysis**

The phase plane analysis presented in Fig. 7 is standard visualization of dynamics in a system of 2 variables. In general, phase space is the space of these variables (i.e. $U_{On}$ and $U_{Off}$). Since the variables are plotted against each other, the graph shows their interdependence. As the variables change in time, they draw a trace in the phase space called a trajectory. The curves defined as $dX/dt=0$, where $X$ is one of the variables, are called nullclines. They show locations in the phase space where one of the variables does not change (the trajectory has zero slope, hence the name). Intersections of the nullclines determine points where both variables do not change $\frac{dU_{On}}{dt}=0,\frac{dU_{Off}}{dt}=0$. At this location, called equilibrium states, all variations stop. Depending on the way the nullclines intersect (Fig. 7), the equilibria can be stable (attracting), unstable (repelling), or saddle. For example, there are 2 stable (at the axis) and 1 saddle (middle) equilibrium states in all three panels of Fig. 7. Depending on initial conditions, a trajectory can be attracted to one of the stable equilibria. The saddle has the role of separating the flows to stable equilibrium states, as the trajectories shows (Fig. 7 grey).

The system of 5 variables Eqns. (1-5) has been further reduced for phase plane analysis. The values of their BLA inputs are assumed to be fixed at approximate levels reached in extinction for our simulations of naïve, acute, and chronic alcohol conditions (Figs. 3-5A). Specifically, in the naïve case $U_{BAf}=0.15, U_{BAe}=0.9, U_{LA}=0.8$; in the acute case $U_{BAf}=0.1, U_{BAe}=0.95, U_{LA}=1$, and in the chronic case, these values are 0.4, 1 and 1 respectively. The noise was removed from the model to emphasize the dynamics. The nullclines of the resulting system are defined by

$$\frac{dU_{On}}{dt}=0 \Rightarrow F\left( W_{BAf\to CeA}*U_{BAf}+W_{LA\to CeA}*U_{LA}-W_{CeAinhib}*U_{Off}+{Dr}_{CeA} \right)-U_{On}=0$$

$$\frac{dU_{Off}}{dt}=0 \Rightarrow F\left( W_{BAe\to CeA}*U_{BAe}-W_{CeAinhib}*U_{On}+{Dr}_{CeA} \right)-U_{Off}=0$$

Thus, these nullclines directly represent the CeA neuron response curves (Fig. 7):

$$U_{On}=F\left( W_{BAf\to CeA}*U_{BAf}+W_{LA\to CeA}*U_{LA}-W_{CeAinhib}*U_{Off}+{Dr}_{CeA} \right)$$

$$U_{Off}=\left( W_{BAe\to CeA}*U_{BAe}-W_{CeAinhib}*U_{On}+{Dr}_{CeA} \right)$$

These simplifications are validated by simulations of the original 5-variable model showing that its trajectory is attracted to a vicinity of a stable equilibrium state, though it keeps wandering around it due to noise (Fig. 7 grey).

**Sex Differences and Implications for Fear Conditioning in Alcohol Exposure**

Sex is a critical factor influencing fear learning, extinction, and reinstatement (Day and Stevenson, 2020). Females exhibit enhanced fear extinction compared to males under normal conditions, potentially due to hormonal enhancement of amygdala-prefrontal interactions (Graham and Milad, 2013). Amygdalar synaptic plasticity is heavily influenced by sex hormones (Carvalho-Netto et al., 2011)., and amygdala interactions with mPFC and hippocampus differ in males and females (Zeidan et al., 2011). Additionally, females are more susceptible to stress effects on development of psychopathology, including AUD (Armstrong et al., 2018). Our model provides a tool to mechanistically connect sex- and alcohol-related modulations in synaptic plasticity and amygdala connectivity to further dissect their impacts on fear conditioning. Such adaptations may both help disentangle sex-dependent mechanisms in fear conditioning and aid in identifying sex-specific therapeutic targets for comorbid AUD and PTSD.

Sex differences further expand to the extended amygdala, where the bed nucleus of the stria terminalis (BNST) is implicated in anxiety-like behavior and conditioned responses to diffuse and/or unpredictable threats (Urien and Bauer, 2022). Incorporating the BNST into our model could be a straightforward expansion as both the BLA and CeA interact with the BNST. It would expand the framework of this model to anxiety-like behaviors and their interaction with AUD.

**Comparison with earlier models**

Importantly, our model allows for expansion to a more complex amygdala structure not possible with earlier models. One previous model (Carrere and Alexandre, 2015). arrived at a very similar model structure, but their arguments were mostly functional. Consequently, that model could not rigorously calibrate the circuit using electrophysiological data, whereas ours does. Finally, we have de novo built in the influence of alcohol. While models exist that integrate more advanced neurobiological data, they represent only a subset of the amygdala circuitry (Cattani et al., 2023; Feng et al., 2016; John et al., 2016; Li et al., 2009; Neilan et al., 2021; Vlachos et al., 2011) or employed a circuit design contradicted by later experiments (Bennett et al., 2019; Krasne et al., 2011). Those models may be suitable for specific experiments, but they sacrifice important distinctions in contributions of basal, lateral, and central amygdala through over-reduction of the system. For example, the extinction impairments displayed in acute and chronic alcohol have distinct mechanisms rooted in BLA and CeA, respectively.

**Additional references:**

Armstrong JL, Ronzitti S, Hoff RA, Potenza MN (2018) Gender moderates the relationship between stressful life events and psychopathology: Findings from a national study. J Psychiatr Res 107:34–41.

Bennett MR, Farnell L, Gibson WG, Lagopoulos J (2019) A model of amygdala function following plastic changes at specific synapses during extinction. Neurobiol Stress 10:100159.

Carrere M, Alexandre F (2015) A pavlovian model of the amygdala and its influence within the medial temporal lobe. Front Syst Neurosci 9.

Carvalho-Netto EF, Myers B, Jones K, Solomon MB, Herman JP (2011) Sex differences in synaptic plasticity in stress-responsive brain regions following chronic variable stress. Physiol Behav 104:242–247.

Cattani A, Arnold DB, McCarthy M, Kopell N (2023) Basolateral amygdala oscillations enable fear learning in a biophysical model. eLife 12.

Ciocchi S, Herry C, Grenier F, Wolff SBE, Letzkus JJ, Vlachos I, Ehrlich I, Sprengel R, Deisseroth K, Stadler MB, Müller C, Lüthi A (2010) Encoding of conditioned fear in central amygdala inhibitory circuits. Nature 468:277–282.

Day HLL, Stevenson CW (2020) The neurobiological basis of sex differences in learned fear and its inhibition. Eur J Neurosci 52:2466–2486.

Feng F, Samarth P, Paré D, Nair SS (2016) Mechanisms underlying the formation of the amygdalar fear memory trace: A computational perspective. Neuroscience 322:370–376.

Graham BM, Milad MR (2013) Blockade of estrogen by hormonal contraceptives impairs fear extinction in female rats and women. Biol Psychiatry 73:371–378.

John YJ, Zikopoulos B, Bullock D, Barbas H (2016) The Emotional Gatekeeper: A Computational Model of Attentional Selection and Suppression through the Pathway from the Amygdala to the Inhibitory Thalamic Reticular Nucleus. PLOS Comput Biol 12:e1004722.

Krasne FB, Fanselow MS, Zelikowsky M (2011) Design of a Neurally Plausible Model of Fear Learning. Front Behav Neurosci 5.

Li G, Nair SS, Quirk GJ (2009) A Biologically Realistic Network Model of Acquisition and Extinction of Conditioned Fear Associations in Lateral Amygdala Neurons. J Neurophysiol 101:1629–1646.

Neilan RM, Majetic G, Gil-Silva M, Adke AP, Carrasquillo Y, Kolber BJ (2021) Agent-based modeling of the central amygdala and pain using cell-type specific physiological parameters. PLOS Comput Biol 17:e1009097.

Urien L, Bauer EP (2022) Sex Differences in BNST and Amygdala Activation by Contextual, Cued, and Unpredictable Threats. eNeuro 9.

Vlachos I, Herry C, Lüthi A, Aertsen A, Kumar A (2011) Context-Dependent Encoding of Fear and Extinction Memories in a Large-Scale Network Model of the Basal Amygdala. PLoS Comput Biol 7:e1001104.

Zeidan MA, Igoe SA, Linnman C, Vitalo A, Levine JB, Klibanski A, Goldstein JM, Milad MR (2011) Estradiol modulates medial prefrontal cortex and amygdala activity during fear extinction in women and female rats. Biol Psychiatry 70:920–927.
